# Lead-Halide Scalar Couplings in ^207^Pb NMR of APbX_3_ Perovskites (A = Cs, Methylammonium, Formamidinium; X = Cl, Br, I)

**DOI:** 10.1038/s41598-020-65071-4

**Published:** 2020-05-19

**Authors:** Marcel Aebli, Laura Piveteau, Olga Nazarenko, Bogdan M. Benin, Franziska Krieg, René Verel, Maksym V. Kovalenko

**Affiliations:** 1 Department of Chemistry and Applied Biosciences, ETH Zürich, Vladimir-Prelog-Weg 1-5, CH-8093 Switzerland; 2 Empa-Swiss Federal Laboratories for Materials Science and Technology, Dübendorf, Überlandstrasse 129, CH-8600 Switzerland; 3 Present Address: Conditions Extrêmes et Matériaux: Haute Température et Irradiation (CEMHTI), UPR 3079 CNRS, Université d’Orléans, 1D Avenue de la Recherche Scientifique, 45071 Orléans, France

**Keywords:** Chemistry, Inorganic chemistry

## Abstract

Understanding the structure and dynamics of newcomer optoelectronic materials - lead halide perovskites APbX_3_ [A = Cs, methylammonium (CH_3_NH_3_^+^, MA), formamidinium (CH(NH_2_)_2_^+^, FA); X = Cl, Br, I] - has been a major research thrust. In this work, new insights could be gained by using ^207^Pb solid-state nuclear magnetic resonance (NMR) spectroscopy at variable temperatures between 100 and 300 K. The existence of scalar couplings ^1^J_Pb-Cl_ of *ca*. 400 Hz and ^1^J_Pb-Br_ of *ca*. 2.3 kHz could be confirmed for MAPbX_3_ and CsPbX_3_. Diverse and fast structure dynamics, including rotations of A-cations, harmonic and anharmonic vibrations of the lead-halide framework and ionic mobility, affect the resolution of the coupling pattern. ^207^Pb NMR can therefore be used to detect the structural disorder and phase transitions. Furthermore, by comparing bulk and nanocrystalline CsPbBr_3_ a greater structural disorder of the PbBr_6_-octahedra had been confirmed in a nanoscale counterpart, not readily captured by diffraction-based techniques.

## Introduction

Semiconducting lead halide perovskite materials, foremost of APbX_3_-type [A = Cs, methylammonium (CH_3_NH_3_^+^, MA), formamidinium (CH(NH_2_)_2_^+^, FA); X = Cl, Br, I], have raised tremendous interest over the past years due to their outstanding optoelectronic properties, which find application in solar cells^1,2^, X-ray^3^ and gamma detectors^4–6^ and light-emitting devices^7–14^. These semiconductors exhibit unusually high defect-tolerance, which is the nearly intrinsic semiconducting behaviour in spite of the high abundance of structural imperfections. Such defect-tolerance had been attributed to the specifics of the electronic structure, crystal structure and structural dynamics^15–21^. It is therefore fundamental to develop an experimental toolset and a related mind-set for studying the local structure and structural dynamics as well as their relationship to the electronic and physical properties of these semiconductors. Solid-state nuclear magnetic resonance (NMR) is a powerful technique for characterizing solid materials. It is complementary to X-ray diffraction, as it is particularly sensitive to the local environment of nuclei. Chemical composition of APbX_3_ makes these compounds very well suited for NMR, owing to the range of NMR-active nuclei (^1^H^22–25^, ^2^H^22,23,26–28^, ^13^C^22–25,29^, ^14^N^22–27,29^, ^15^N^25,30^, ^133^Cs^29,31^, ^207^Pb^23–25,27,31–33^, ^35^Cl, ^37^Cl, ^79^Br, ^81^Br, ^127^I)^34^. In this contribution, we focus on ^207^Pb NMR spectroscopy of APbX_3_ compounds and report on the existence of scalar lead-halide J-couplings in some of them. ^1^J_Pb-Cl_ of *ca*. 400 Hz and ^1^J_Pb-Br_ of *ca*. 2.5 kHz have been measured for MAPbX_3_ and CsPbX_3_ compounds. For other compounds within the APbX_3_ family, scalar couplings are elucidated to be on the order of 2–3 kHz but had not been spectrally resolved. The temperature dependence of the couplings correlates with the known reduction of the structural dynamics and ionic mobility in these perovskites^35^.

**Figure 1:**
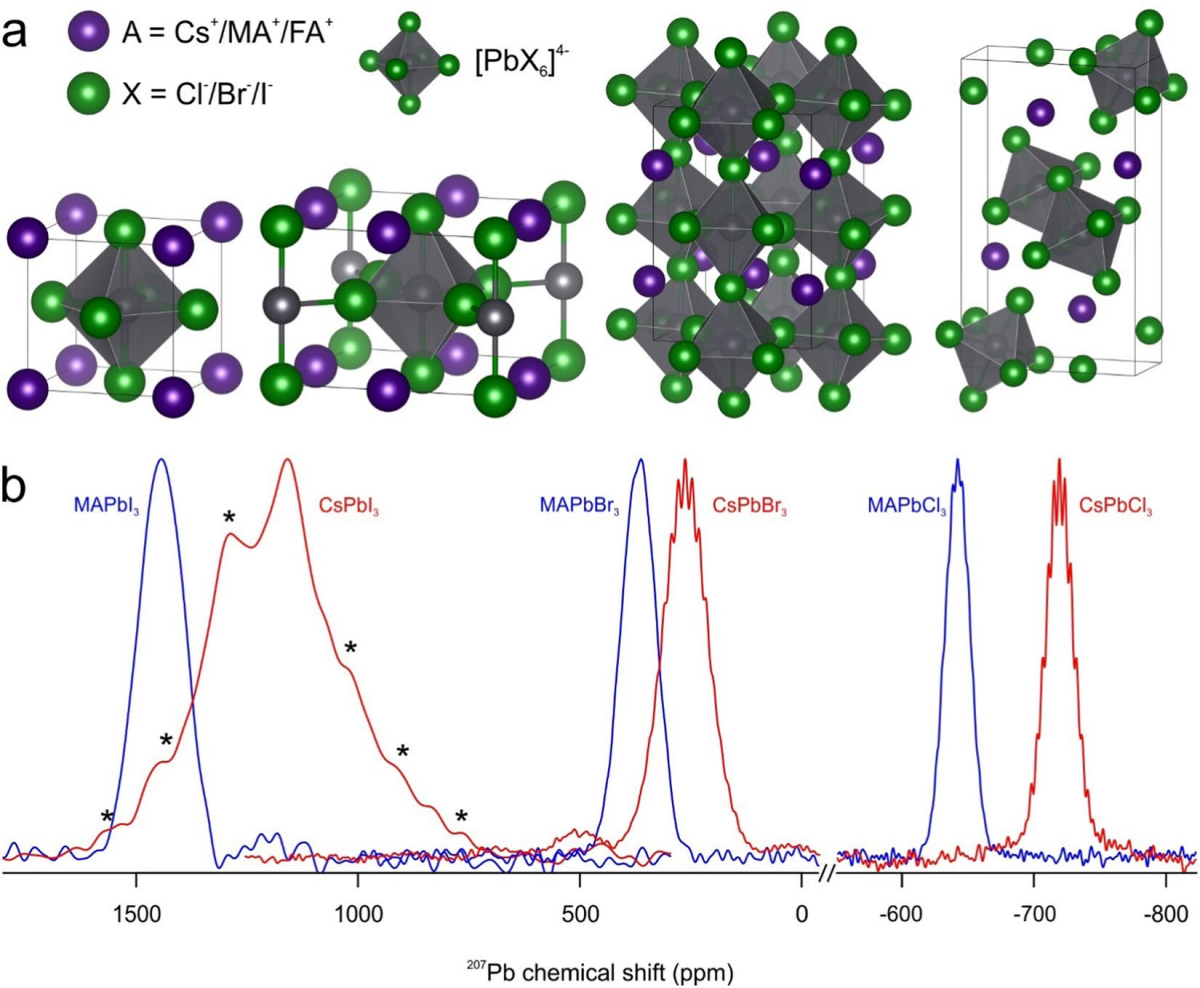
(**a**) Cubic, tetragonal and orthorhombic structures of 3D-perovskites as well as the 1D-structure of orthorhombic CsPbI_3_. (**b**) ^207^Pb NMR spectra of MAPbI_3_, CsPbI_3_, MAPbBr_3_, CsPbBr_3_, MAPbCl_3_ and CsPbCl_3_. The spectra of MAPbI_3_, CsPbI_3_, MAPbBr_3,_ and CsPbBr_3_ were acquired at 16.4 T and the spectra of MAPbCl_3_ and CsPbCl_3_ were acquired at 11.7 T at room temperature (RT) using powdered materials. Spinning side bands are marked by asterisks.

In APbX_3_ perovskite compounds, corner-sharing lead-halide octahedra form a 3-dimensional (3D) anionic network, charge-stabilized by A-cations filling large 12-fold-coordinated voids in-between the octahedra. Several 3D-polymorphs of these compounds exist, but they differ in the distortion of the lead-halide octahedral lattice (Fig. 1a; see Table S1 for a detailed overview of known structures at various temperatures). This structural data is correlated in the following discussion with our NMR data. The compounds consist of a dynamic inorganic PbX-framework with a high concentration of point defects (higher than 0.4% in MAPbI_3_ at room temperature^36^) leading to defect-mediated hopping of the halide anions^37^. These frameworks are coupled through ion-ion interactions and hydrogen bonds to the A-cation. The rotation and displacement of the A-cations lead to distortions and anharmonic vibrations in the whole perovskite structures^38^, which has been shown by Whalley *et al*.^39^ and Beecher *et al*.^40^ for MAPbI_3_ and by Marronnier *et al*.^41,42^ for CsPbI_3_. All these dynamic processes take place in the picosecond time scale and contribute to the soft structure of the lead halide perovskites. They can be diminished or for some rotations even completely suppressed by reducing the temperature.

NMR studies on lead halide perovskites had already been launched in the 1980s. For MAPbX_3_, Wasylishen reported in 1985 on the dynamics of the organic cation and phase transitions using ^2^H and ^14^N NMR^26^. Most of the subsequent studies concentrated on ^1^H, ^2^H, ^13^C, ^14^N, ^15^N nuclei to characterize and understand the dynamics and mobility of the organic cations (MA or FA)^22–27,43–45^. Only a few studies concerned ^133^Cs NMR^29,31,46–51^. As to the halides, NMR spectroscopy had thus far been hampered by their large quadrupolar constants, leading to massively broadened signals and distorted line shapes^52,53^. For this reason, halides are more commonly assessed with nuclear quadrupole resonance (NQR) spectroscopy^25,44,54–58^. Sharma *et al*.^59^ (1987) and the dissertation by Ullmann (1998)^60^ are, to our knowledge, the first reports on ^207^Pb NMR of lead-halide perovskites. Two decades later, ^207^Pb NMR studies had been resumed by Rosales *et al*.^33^, who studied mixed-halide methylammonium perovskites. The last three years have seen the increasing use of ^207^Pb NMR for the characterization of APbX_3_ perovskites and novel lead halide compounds^23–25,27,44,45,61–65^. In these studies, MAPbI_3_ has been the main focus. It was studied at variable temperatures^24,25,27,44^, during decomposition^61^, with dimethylammonium incorporation^45^ and also with bromine substitution^23^. 2D NMR and dynamic nuclear polarization (DNP) NMR was measured for micro- and nanocrystalline MAPbX_3_ at 100 K, which resulted in an enhancement factor of up to 20 for MAPbCl_3_^62,65^. MAPbCl_3_ was additionally measured at various temperatures and its utility as an internal thermometer was shown based on the temperature dependence of its ^207^Pb NMR chemical shift^63^.

## Results and Discussion

Of fundamental importance are the first observations of lead-halide J-coupling in MAPbCl_3_ (^1^J_Pb-Cl_) and in CsPbBr_3_ as well as Cs_4_PbBr_6_^27,32^. J-couplings are mediated through bonds by hyperfine interactions between the nuclei and their local electrons. The J-coupling contains information about bond length and angles. It resonates with the notion that the Pb-halide framework in APbX_3_ compounds exhibits substantial covalency and directionality in the Pb-X bonding, which is needed for efficient through-bond J-coupling^66^. The existence of these bonds has recently been verified with band structure calculations by Goesten and Hoffmann^67^.

Lead-halide J-couplings had already been postulated by Dybowski *et al*. for PbI_2_ (built from face-sharing Pb-I octahedra), but could not be resolved^68^. For the ^207^Pb NMR signal with a full-width at half-maximum (FWHM) of 20 kHz, they calculated a scalar coupling ^1^J_Pb-I_ of 4.9 kHz, which is of a similar magnitude as other scalar couplings involving ^207^Pb^69^.

The observation of J-coupling depends on several factors such as nuclear spin and quadrupolar moment of the halide as well as any dynamic changes in the structure or structural defects. The nuclear spin of the halide determines the number of lines present within the coupling pattern, while the large quadrupolar constant of the halides generally broadens the signal and therefore masks the J-coupling^70^. The next factor that affects the observation of J-coupling is a combined effect of structural inhomogeneities that are primarily due to structural dynamics but also to static structural defects. A distribution of chemical sites, which can be caused, for instance, by vacancies or doping, leads to (inhomogeneous) broadening of the lines. Structure dynamics of the lead halide sublattice falls in the picosecond range^35,71^, which is too fast for the NMR time scale (µs-to-seconds) and will be averaged out and seen as a quasi-static impact on the observed NMR spectrum. The major type of Pb-halide atomic motion is the tilting of the lead-halide octahedra with respect to each other^72,73^. This constantly changes the Pb-X-Pb bond angle and therefore affects the orbital overlap thus obscuring the J-coupling. We therefore expect that the J-coupling is a sensitive probe for the structural dynamics or disorder. This disorder was also related to the phonon anharmonicity observed in hybrid perovskites^39^. It is also important to note that the dynamic disorder of the A-site cation is correlated to that of the Pb-halide framework^38^. Differences in the Pb-X bond length will result in slightly unequal coupling strengths to the individual halides in the lattice^74^. A perfect PbX_6_-octahedron should therefore yield narrower lines compared to a distorted one. This has been shown on ^207^Pb-^19^F couplings in amorphous Pb_5_Ga_3_F_19_^75^. J-couplings to quadrupolar nuclei are known to be self-decoupled, due to their fast relaxation induced by the quadrupole. The fact that in these perovskite materials the couplings are clearly visible, indicates a relatively slow quadrupolar relaxation of the halides^76^. Once observed, the J-coupling could be, in principle, correlated to the atomic structure. While calculations of J-couplings are sufficiently accurate for light elements (^1^H, ^13^C *etc*.) and are generally possible for solid-state inorganic materials^77,78^, there is no such theoretical work yet for lead-halide perovskites. At present, a broader experimental survey over diverse lead halide structure is needed to start drawing correlations with the structural motives (bond length/angles, corner/edge/face-sharing connectivity, octahedral or other lead halide building blocks *etc*.).

All studied APbX_3_ compounds were synthesized using solution-phase methods, as reported earlier (see Supporting Information for synthesis details)^64,79,80^. We note that the as-synthesized 3D-polymorph of FAPbI_3_ (α-phase) rapidly converts into a face-sharing 1D-polymorph (δ-phase) under magic angle spinning (MAS) NMR and therefore we could acquire the spectrum only under static conditions (Fig. S1)^64^. For CsPbI_3_, only the 1D-phase (δ-phase) with edge-sharing octahedra is stable at RT^81,82^. CsPbBr_3_ NCs were synthesized using colloidal methods with long-chain zwitterionic surfactants as surface capping ligands^83^. The NCs were precipitated using antisolvents and properly purified before being isolated by centrifugation, dried under vacuum, and measured as a pure solid.

**Figure 2:**
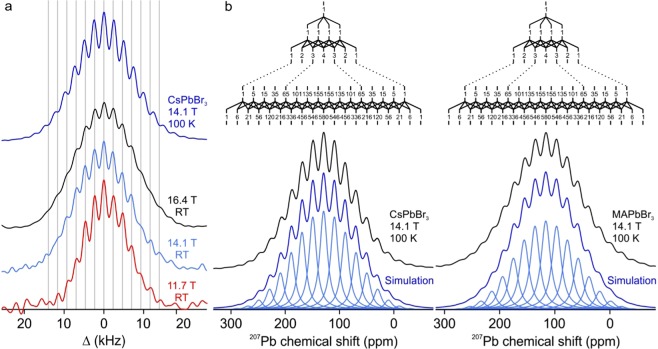
(**a**) ^207^Pb NMR spectra of CsPbBr_3_ acquired at 14.1 T at 100 K (dark blue), 16.4 T at RT (black), 14.1 T at RT (light blue) at 11.7 T and RT (red). The grey lines serve as a guide to the eye by marking the lines of the coupling pattern. The spectra are displayed against a frequency axis centered around 0 Hz. This illustrates most clearly the constant splitting distance (in Hz) between lines, due to a scalar coupling of ^1^J_Pb-Br_ = 2.3 kHz at RT. (**b**) Coupling trees for six equivalent spins I = ^3^/_2_ (top) and the ^207^Pb NMR spectra of CsPbBr_3_ (left) and MAPbBr_3_ (right) acquired at 100 K on a 14.1 T instrument. The acquired spectra are shown in black. The simulated individual lines with intensities obtained from the coupling trees and scalar couplings of 2.5 and 2.4 kHz for CsPbBr_3_ and MAPbBr_3_ respectively are displayed in light blue. The sum of the lines is shown in dark blue and is matching the experimental data.

RT MAS solid-state ^207^Pb NMR spectra of powdered Cs and MA compounds are displayed in Fig. 1b. In agreement with the earlier reports, no scalar couplings were found neither for MAPbI_3_ (blue left, Fig. 1b) nor for MAPbBr_3_ (blue middle, Fig. 1b)^23,24,33,44,59,61,62^. δ-CsPbI_3_ showed no scalar coupling either (red left, Fig. 1b). However, chloride compounds exhibit pronounced J-couplings of ^1^J_Pb-Cl_ = 400 Hz in CsPbCl_3_ (red right, Fig. 1b) and ^1^J_Pb-Cl_ = 390 Hz in MAPbCl_3_ (blue right, Fig. 1b). The latter is similar to that reported by Bernard *et al*. earlier^27^. A particularly strong scalar coupling was observed for CsPbBr_3_; ^1^J_Pb-Br_ = 2.3 kHz (red middle, Fig. 1b). This shows that Pb-Br scalar couplings are possible and that the ^207^Pb NMR spectra of CsPbBr_3_ and Cs_4_PbBr_6_ acquired and presented in the dissertation of Ullmann appear to exhibit coupling patterns as well although not named and rationalized as such by the author^60^. The coupling patterns observed for CsPbBr_3_ as well as for CsPbCl_3_ and MAPbCl_3_ (Fig. S2) can be safely attributed to scalar coupling since the splitting between the lines stays unchanged at different magnetic fields but changes with temperature (Fig. 2).

The coupling patterns match expectations for lead coordinated to six equivalent halides in an octahedron. Under 1^st^ order conditions, the signal of a nucleus coupled with n magnetically equivalent nuclei of spin I is a multiplet with 2nI + 1 lines^84^. Accordingly, for six equivalent halides (n = 6) with spin I = ^3^/_2_ (^35^Cl, ^37^Cl, ^79^Br, ^81^Br) a multiplet with 19 lines is anticipated. The intensity of the individual lines is obtained by constructing a coupling tree for a spin I = ^3^/_2_, related to Pascal’s triangle. The coupling pattern of CsPbBr_3_ and MAPbBr_3_ at 100 K were simulated by using the experimental scalar couplings of 2.5 and 2.35 kHz, the theoretical intensities from the coupling tree to six equivalent spins I = ^3^/_2_ and a Lorentzian line shape with a FWHM of 2.1 and 2.35 kHz. Experimental data and simulations are in excellent agreement (Fig. 2).

Based on the observation that CsPbBr_3_ and MAPbBr_3_ possess similar total linewidth in their ^207^Pb NMR spectra at RT, the hypothesis that an underlying scalar coupling is responsible for the observed overall spectral width is plausible. The absence of visible lines in the multiplet in MAPbBr_3_ can be explained by the greater broadening of the individual lines. Potential interactions causing such line-broadening in NMR are the dipolar coupling, the chemical shift anisotropy (CSA), site disorder, structural dynamics, and fast relaxation^84^. Since the sample is spun at the magic angle at 10 kHz or faster, dipolar couplings (80–280 Hz for Pb-X, and 25 Hz for Pb-Cs and Pb-Pb) and CSAs are averaged out^85^. In a reference experiment, under static conditions, these two interactions also broaden the lines in the ^207^Pb NMR spectrum of CsPbBr_3_ to such an extent that they cannot be resolved anymore (Fig. S3). Hence, we conclude that the site-disorder, dynamics or fast relaxation are greater contributors to the line-broadening in MAPbBr_3_ and MAPbI_3_ than they are in CsPbBr_3_, this way obscuring the J-coupling.

**Figure 3:**
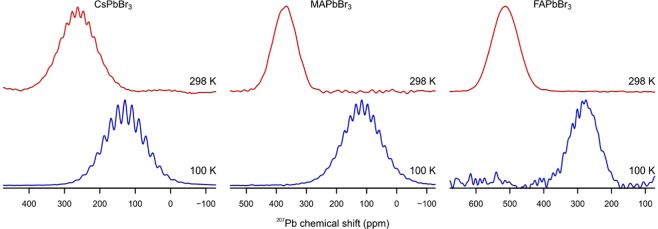
^207^Pb NMR spectra of CsPbBr_3_ (left), MAPbBr_3_ (middle) and FAPbBr_3_ (right). The spectra acquired at RT are shown in red (top) and the ones at 100 K in blue (bottom). The isotropic chemical shifts and the coupling constants are listed in Table 1. A possible coupling in FAPbBr_3_ is difficult to observe due to the low signal-to-noise ratio.

Structural disorder, especially of a dynamic nature, is strongly dependent on temperature in the mobile lead halide lattices as is evidenced by the NMR experiments at low temperatures (Figs. 2 and 3). The low-temperature measurements in this study were carried out at 100 K, since at this temperature all lead halide perovskites are reported to be in their lowest-temperature polymorph. No further phase-transitions were reported to occur below 100 K.

**Table 1:** ^207^Pb NMR data of APbX_3_ perovskites. Acquired under MAS at a) 20 kHz, b) 10 kHz.

Compound	Temperature	δ_iso_ (ppm)	^1^J_Pb-X_ (Hz)	FWHM lines (Hz)	FWHM total (kHz)
CsPbCl_3_	RT^a)^	−714	400	600	2.4
CsPbBr_3_	RT^a)^	262	2300	2400	13.8
	100 K^b)^	130	2500	2100	16.9
CsPbBr_3_ NCs	RT^a)^	252	—	—	17.6
CsPbI_3_	RT^a)^	1160	—	—	20.0
	100 K^b)^	990	—	—	25.0
MAPbCl_3_	RT^a)^	−630	390	600	2.3
MAPbBr_3_	RT^a)^	365	—	—	13.6
	100 K^b)^	125	2350	2350	16.6
MAPbI_3_	RT^a)^	1445	—	—	17.6
	100 K^b)^	1030	—	—	20.0
FAPbBr_3_	RT^a)^	515	—	—	13.1
	100 K^b)^	280	—	—	13.4
FAPbI_3_	RT^a)^	1515	—	—	22.2

Spinning at faster MAS increases the temperature of the sample and therefor changes the chemical shift (Fig. S3).

The isotropic chemical shift of all compounds substantially changes towards higher frequencies upon cooling, which is typical for the ^207^Pb NMR signal. For CsPbBr_3_, one can clearly observe an improved resolution of the coupling pattern at 100 K (Fig. 3). The FWHM of the individual lines narrows from 2.7 to 2.1 kHz while the coupling strength increases from 2.3 to 2.5 kHz. The stronger coupling can be explained by the contraction of the unit cell that occurs upon cooling leading to a shortening of the Pb-Br bonds^86–88^. A more prominent improvement of the coupling resolution can be observed for MAPbBr_3_. After the phase transition to the orthorhombic phase below 150 K the coupling pattern appears (Figs. 3 and S4). In this phase, the motion of the MA-cation is limited to rotation around its C-N axis. This has been shown by Wasylishen *et al*.^26^ with ^2^H and ^14^N NMR, by Zhu *et al*.^35^ where the time-resolved optical Kerr effect at various temperatures was measured and calculated with molecular dynamics simulations by Even *et al*.^89^. In its high-temperature phases, the MA cations can be described as an anisotropic molecular liquid while at 77 K it was found to be frozen. The dynamics of the A-cation and the halides are coupled by hydrogen bonds^37^. A mobile cation with rotational freedom will, therefore, lead to a higher distortion of the PbX_6_-octahedra. These distortions are so far only visible in synchrotron methods like total scattering or pair distribution function analysis^90^. In NMR these deformations of the octahedra will result in a smeared out coupling pattern. ^207^Pb NMR is, therefore, a useful tool to indirectly detect A-cation dynamics and its induced distortion of the inorganic lattice.

For FAPbBr_3_, no coupling was observed despite cooling, and the total FWHM does not change significantly. This indicates a still highly mobile and disordered surrounding for the ^207^Pb nuclei. Indeed, it has been shown by single-crystal XRD that the bromides have large displacement factors orthogonal to the Pb-Br bonds even at 100 K^91^. Additionally, by synchrotron XRD, four distinct positions for Br where detected^92^. This displacement, therefore, leads to inhomogeneously broadened lines and obscures the coupling pattern. A fast deformation dynamics in hybrid organic inorganic perovskites was also observed by measuring the hot fluorescence emission^35^. This deformation was attributed to the coupling of the liquid-like motion of the organic cations with the inorganic framework. With ^207^Pb NMR we could also detect these distortions, present even at 100 K (Fig. 3), making it a powerful method to probe for lattice dynamics at various temperatures. The signal to noise ratio for the low temperature measurement is lower compared to CsPbBr_3_ and MAPbBr_3_ measurements, due to the lower number of scans.

The lines of APbBr_3_ are several times broader than their chlorine analogs (2500 Hz vs. 600 Hz). Both bromine and chlorine possess two isotopes with high natural abundance% ^35^Cl (76%) and ^37^Cl (24%) as well as ^79^Br (51%) and ^81^Br (49%). The J-couplings between ^207^Pb and the different isotopes further contribute to the broadening of the individual lines in the coupling pattern, which cannot be quantified at this stage. Another possible contribution to the line broadening could be faster relaxation due to the close vicinity of ^207^Pb to quadrupoles (halides). All isotopes of chlorine and bromine have a spin of I = ^3^/_2_, but the quadrupole moments of the bromine isotopes are four times larger than the ones of the chlorine. This effect should be even more severe for iodine in APbI_3_ perovskites, with a spin of I = ^5^/_2_ and a quadrupole moment more than twice as large as the bromine. Possible Pb-I couplings were calculated by Dybowski *et al*. in PbI_2_ to be around 4.9 kHz and by Bernard *et al*. in MAPbI_3_ to be between 2 and 3 kHz. So far, none of these couplings could be experimentally resolved.

For MAPbI_3_, low activation energy for migration of iodine and MA was calculated by Eames *et al*.^36^, and confirmed by experimental studies^24,93^. This ion migration will additionally disturb the PbI_6_-octahedra. Low-temperature measurements will, therefore, be indispensable for the resolution of Pb-I scalar couplings. At 100 K, a broad tensor was observed for APbI_3_ (Fig. S5). The isotropic chemical shifts are 990 ppm for CsPbI_3_ and 1030 ppm for MAPbI_3_ with FWHM values of around 25 and 20 kHz, respectively. The presence of spinning side bands is an indicator for high anisotropy around the lead nuclei. The spinning side bands overlap complicating the identification of an eventual coupling pattern. Higher spinning speeds would be required to prevent an overlap of the spinning side bands and help resolve the coupling pattern.

**Figure 4:**
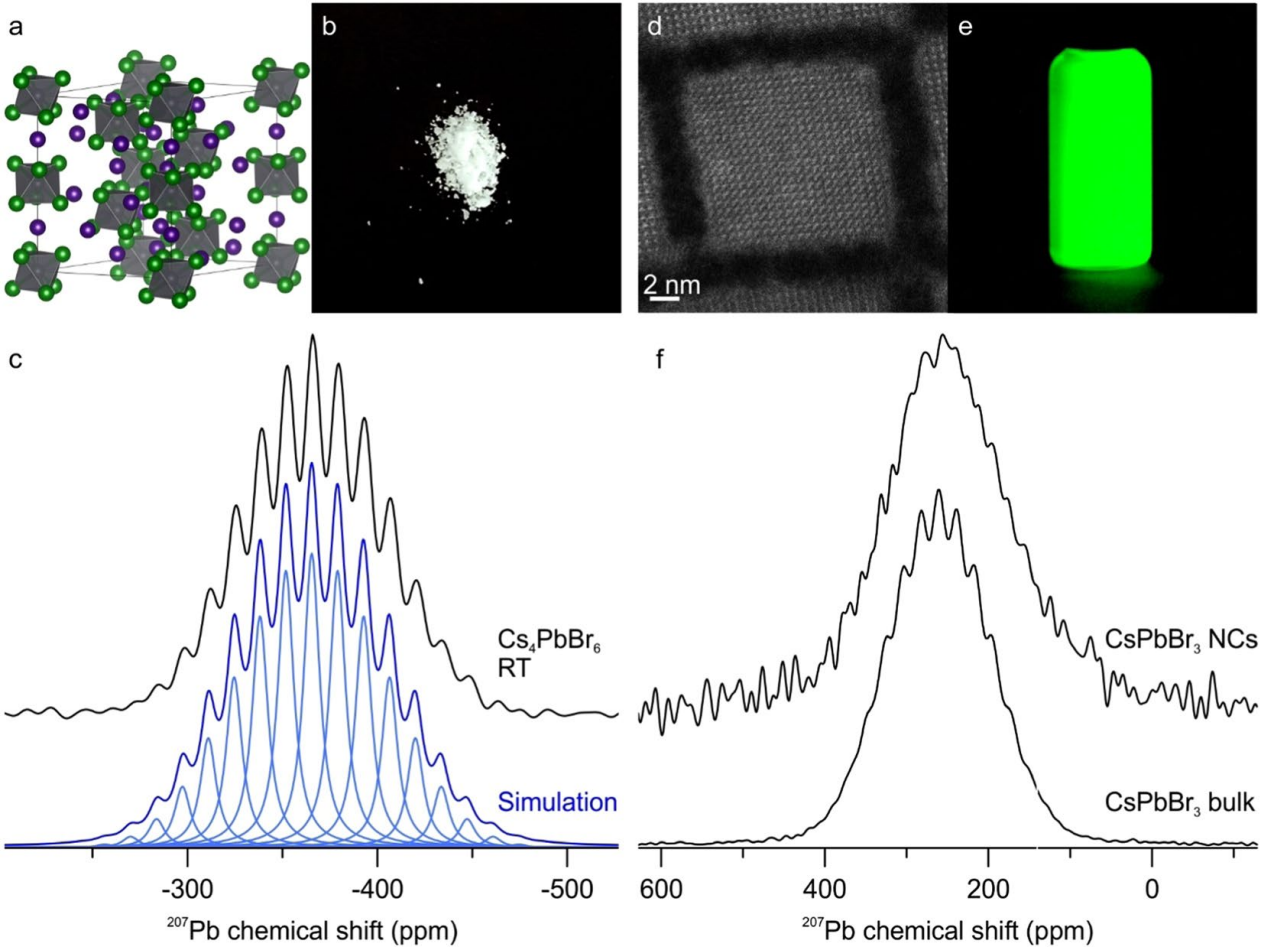
(**a**) Crystal structure of 0D Cs_4_PbBr_6_ with isolated PbBr_6_-octahedra. (**b**) Picture of Cs_4_PbBr_6_ powder. (**c**) ^207^Pb NMR spectrum of Cs_4_PbBr_6_ (black) at RT acquired on a 16.4 T spectrometer. The simulated individual lines with intensities obtained from the coupling tree, a FWHM of 1.5 kHz and a scalar coupling of 2.0 kHz are displayed in light blue. The sum of the lines is shown in dark blue and is matching the experimental data. (**d**) High-Angle Annular Dark-Field Scanning Transmission Electron Microscopy (HAADF-STEM) image of a single CsPbBr_3_ NC. (**e**) Colloidal solution of CsPbBr_3_ NCs under UV excitation. (**f**) ^207^Pb NMR spectra of CsPbBr_3_ bulk (bottom) and NCs (top) acquired at RT on a 11.7 T instrument with identical measurement and processing parameters. The coupling pattern cannot be resolved for the NCs and the total line width increases to 17.6 kHz. This was attributed to higher PbBr_6_-octahedral disorders over the whole nanocrystal compared to the bulk material.

Since lead halide couplings were observed for several 3D-perovskite bromides and chlorides, we have looked for the occurrence of ^1^J_Pb-X_ couplings in other octahedrally coordinated lead halides, such as the 0D Cs_4_PbBr_6_. Here too, we could observe a well-resolved coupling of 2.0 kHz was detected even at RT (Fig. 4). The FWHM is 1.5 kHz, which is significantly narrower than that of CsPbBr_3_ at 2.4 kHz. All Pb-Br bonds are identical in Cs_4_PbBr_6_ unlike in CsPbBr_3_, and this leads to a smaller coupling constant distribution that narrows the linewidth^94^. This clearly shows the effect of lattice distortions on the coupling. For a better insight into this effect, the comparison with 0D hybrid materials with MA and FA would be indispensable. Unfortunately, these materials are so far not known.

We have then also probed the effect of dimensionality by comparing bulk material and colloidal nanocrystals (NCs) of CsPbBr_3_ (Fig. 4). These NCs have recently become an object of intense research due to their outstanding luminescent properties – narrow-band emission with high absolute quantum yields, highly suited for applications in television displays^8,21,83^. The coupling cannot be resolved at RT, and the total linewidth increases to 17.6 kHz. This is attributed to higher disorder and higher ion-mobility in NCs. In fact, this result correlates well with the model proposing coherent twins and dynamic disorder in these nanocrystals from the analysis of X-ray total scattering techniques and the Debye scattering equation^95^.

## Conclusions

In summary, scalar couplings between ^207^Pb and halide nuclei (^35^Cl, ^35^Cl,^79^Br, ^81^Br) have been detected in ^207^Pb NMR spectra of APbX_3_ perovskites. The coupling strengths are in the range of 400 Hz for ^1^J_Pb-Cl_ and 2.3 kHz for ^1^J_Pb-Br_. Only CsPbCl_3_ and CsPbBr_3_ exhibit pronounced coupling patterns at RT. The substantial diminishing of structural dynamics in MAPbBr_3_ at temperatures below 150 K results in the resolution of the J-coupling. For the iodine compounds, a coupling ^1^J_Pb-I_ of *ca*. 3 kHz can only be postulated based on the overall spectral line width, but it could not be experimentally resolved. Future studies might concentrate on resolving Pb-iodide couplings at lower magnetic fields. ^207^Pb NMR has shown to be an easily accessible tool to detect permanent and dynamic distortions in the inorganic framework of perovskites. This shows its great potential to better characterize these materials, which is not possible by normal X-ray diffraction. Another important avenue is to probe the relationship between Pb-Br J-couplings and the structural disorder induced by the dimensionality, for instance, in colloidal CsPbBr_3_ NCs.

## Methods

APbX_3_ (A = Cs, MA, FA; X = Cl, Br, I) compounds were synthesized in the corresponding hydrohalic acid. CsPbX_3_ was additionally prepared from the solid state by melting together CsX and PbX_2_ in a 1:1 ratio. CsPbBr_3_ NCs were prepared by hot injection using long-chain zwitterionic molecules as capping ligands^83^. See SI for further details. The purity of all compounds was confirmed by powder X-ray diffraction (pXRD). All samples were ground into a fine powder and densely packed into ZrO_2_ rotors. Solid-state Magic Angle Spinning (MAS) NMR experiments at ambient conditions were performed on three Bruker Avance IIIHD spectrometers (Bruker Biospin, Fällanden, Switzerland). The 11.7 and 16.4 T instruments were equipped with 2.5 mm two-channel and three-channel solid-state probe heads. The spinning frequency was set between 0 and 20 kHz. The 14.1 T magnet was equipped with a 3.2 mm double-channel MAS probe and a MAS spinning rate of 10 kHz was used. Low-temperature experiments were conducted on the 14.1 T Bruker instrument equipped with a 3.2 mm double-channel low-temperature MAS probe using MAS spinning of 10 kHz. ^207^Pb NMR chemical shifts were referenced to PbMe_4_. A Hahn echo pulse-sequence was used for all measurements with an echo delay between 0.1 and 0.5 ms^96^. The rf field of the echo pulses was set to 35.7, 26.3 and 19.8 kHz at 11.7, 14.1 and 16.4 T, respectively, which is strong enough to be refocused the complete spectra of the 3D phases. The 1D phases are too broad (>140 kHz) to be refocused completely.

## Supplementary information

Supplementary information.
