# Supplementary material for: Lead-Halide Scalar Couplings in 207Pb NMR of APbX3 Perovskites (A = Cs, Methylammonium, Formamidinium; X = Cl, Br, I)
Source: Sci Rep. 2020 May 19;10:8229. doi: 10.1038/s41598-020-65071-4 (PMC7237655; doi:10.1038/s41598-020-65071-4)
Supplement: Supplementary file 1 — Supplementary information. [file 41598_2020_65071_MOESM1_ESM.docx]

**Supporting Information for:**

Lead-Halide Scalar Couplings in ^207^Pb NMR Spectra of APbX_3_ Perovskites

Marcel Aebli,^†‡^ Laura Piveteau,^†‡^ Olga Nazarenko,^†‡^ Bogdan M. Benin,^†‡^ Franziska Krieg,^†‡^ René Verel*^†^ and Maksym V. Kovalenko*^†‡^

^†^Department of Chemistry and Applied Biosciences, ETH Zürich, Vladimir-Prelog-Weg 1-5, CH-8093, Switzerland

^‡^Empa-Swiss Federal Laboratories for Materials Science and Technology, Dübendorf, Überlandstrasse 129, CH-8600, Switzerland

*E-mail: [mvkovalenko@ethz.ch](mailto:mvkovalenko@ethz.ch)

[verelr@ethz.ch](mailto:verelr@ethz.ch)

**1. Materials and Methods**

**Chemicals.**

Lead acetate trihydrate (Pb(OAc)_2_×3H_2_O, ≥99.99%), lead nitrate (Pb(NO_3_)_2_, ≥99.99% trace metals basis), lead iodide (PbI_2_, 99%), lead chloride (PbCl_2_, 99.999%), cesium bromide (CsBr, 99.9%), formamidinium acetate (FAOAc, 99 %), methylamine solution (CH_3_NH_2_, 40% in water), hydrochloric acid (HCl, 37% in water), hydroiodic acid (HI, 57% in water), oleic acid (≥99%), bromine, 1-octadecene, 3-(N,N-dimethyloctadecylammonio)-propanesulfonate (>99.0%) and ethanol (≥99.8%) were purchased from Aldrich; hydrobromic acid (HBr, 48 % water solution), lead bromide (PbBr_2_, 98%), and cesium iodide (CsI, 99.9%) were obtained from Acros; cesium chloride (CsCl, 99.9%) was purchased from ABCR; cesium carbonate was purchased from Fluorochem. Chemicals were used as received without further purification.

The bulk APbX_3_ compounds had been synthesized by adapting the methods from the earlier literature.^1, 2^

**FAPbBr_3_** was crystallized upon combination of hot precursor solutions (~90 °C): PbBr_2_ (0.01 mol) in 4 mL of HBr and FAOAc (0.01 mol) in 1 mL of HBr. The mixture was then cooled down to room temperature (RT). The red crystals were separated by filtration and dried under ambient conditions. Synthesis yield - 85 %.

**CsPbCl_3_** was obtained by dissolving PbCl_2_ (5 mmol) in 7.5 mL of HCl. A solution of CsCl (5 mmol) in 1 mL water was added. The white precipitate was filtered of and washed thrice with EtOH before being dried under reduced pressure. **CsPbBr_3_** and **CsPbI_3_** were synthesized by the same adapted method using the corresponding halide reagents.

**CsPbI_3_** from the solid state was obtained by mixing together in a mortar CsI and PbI_2_ in stoichiometric quantities and sealing under vacuum (10^-3^ mbar) in quartz (12 mm O.D., 1 mm thickness). The quartz ampule was heated to 650 °C at 300 °C/hr and held at this temperature for 1 hr before cooling to 120 °C at 200 °C/hr. The sample was kept at 120 °C for 24 hrs and was then quickly cooled in water. While the product remained black for some time, it partially converted to the yellow, orthorhombic phase before the measurement could be conducted (Figure S7).

**MAPbCl_3_** was obtained by dissolving Pb(NO_3_)_2_ (6 mmol) in 6 mL of HCl. The solution was heated to 80 °C and methylamine (72 mmol) was added. The white precipitate was filtered of and washed thrice with EtOH before being dried under reduced pressure. MAPbBr_3_ and MAPbI_3_ were synthesized by the same adapted method using HBr and HI, respectively.

**CsPbBr_3_** **NCs** were obtained according to our previous report,^3^ as detailed below.

**Cesium oleate (0.4 M):** 1.628 g of Cs_2_CO_3_ (10 mmol, 2 eq. Cs) and 5 mL of oleic acid (16 mmol, 0.8 eq.) were evacuated in a three-neck flask along with 20 mL of ODE at RT until the first gas evolution subsides and was then further evacuated at 25-120 °C for 1 hour.

**Lead (II)-oleate (0.5 M):** 4.6066 g of lead (II) acetate trihydrate (12 mmol, 1 eq.) and 7.6 mL of oleic acid (24 mmol, 2 eq.) were evacuated in a three-neck flask along with 16.4 mL of ODE at RT until the first gas evolution subsides and then further evacuated at 25-120 °C for 1 hour.

**TOPBr_2_ (0.5 M):** TOP (6 mL, 13 mmol) and Br_2_ (0.6 mL, 11.5 mmol) were reacted under inert atmosphere and diluted with toluene (18.7 mL) once the reaction was cooled to RT.

**CsPbBr_3_ Nanocrystals:** Pb-oleate (5 mL, 2.5 mmol), Cs-oleate (4 mL, 1.6 mmol), 3-(N,N-dimethyloctadecylammonio)-propanesulfonate (0.215 g, 0.5 mmol) were mixed with 50 mL 1-octadecene and heated to 120°C under vacuum, where the atmosphere was changed to argon. The temperature was further elevated to 130°C where TOPBr_2_ was injected (5 mL, 5 mmol of halides). The reaction was cooled down immediately by an ice bath.

**Isolation and Purification:** To the crude solution (64 mL) 128 mL of ethyl acetate were added and the NCs were precipitated by centrifugation at 29500 times g (g is the earth gravitation constant) for 10 minutes. The NCs were dispersed in 20 mL of toluene and precipitated with 40 mL of ethyl acetate and centrifugation at 29500 times g for 1 min. During the second and third purification step solvents were reduced by factor of two for each step. After the last precipitation the NCs were dried under vacuum overnight and the powders were measured.

**2. Characterization**

**Powder X-ray diffraction** (pXRD) patterns were collected in transmission mode with a STADI P diffractometer (STOE&Cie GmbH), equipped with a curved Ge (111)-Monochromator (CuK_α1_ = 1.54056Å) and a silicon strip MYTHEN 1K Detector (Fa. DECTRIS). For the measurement, ground powder was placed between adhesive tape.

**3. Additional NMR data**

**
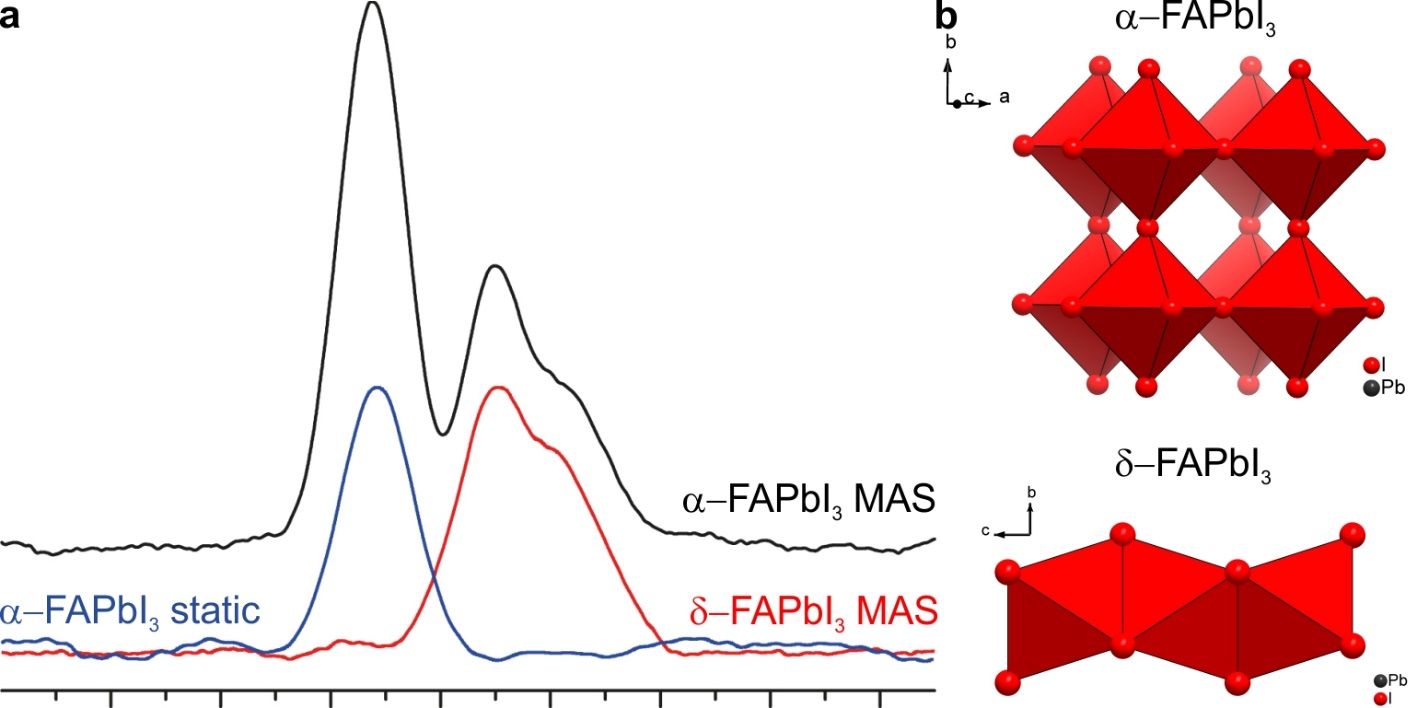
**

**Figure S1**. (**a**) ^207^Pb MAS NMR spectrum of α-FAPbI_3_ (black) showing two different signals. The spectra of α-FAPbI_3_ in static mode (blue) and of δ-FAPbI_3_ (red) with MAS are plotted for comparison. (**b**) Crystal structure of the 3D α-FAPbI_3_ and 1D δ-FAPbI_3_.^4^

**
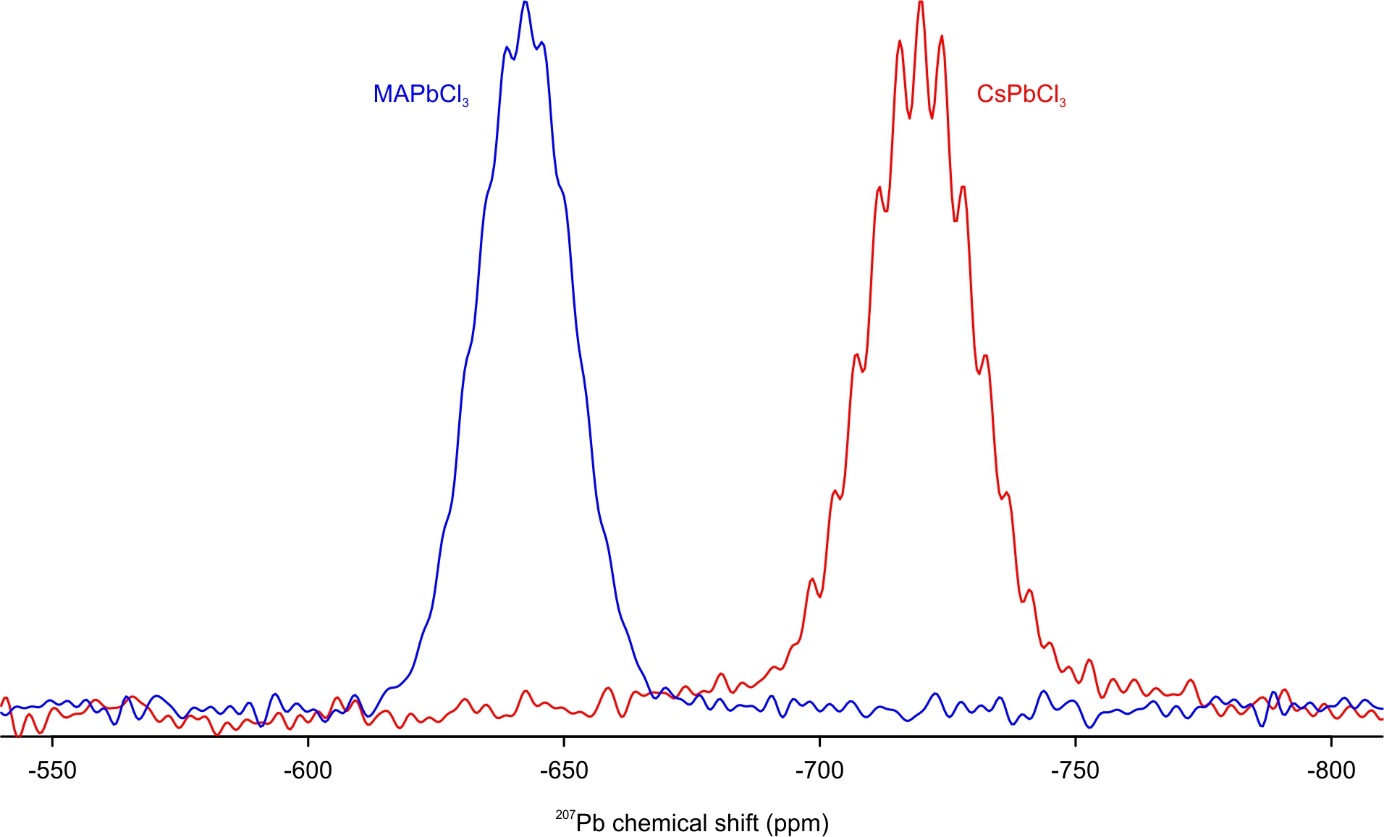
**

**Figure S2**. ^207^Pb MAS NMR spectra of MAPbCl_3_ (blue) and CsPbCl_3_ (red) acquired on an 11.7 T instrument at RT.


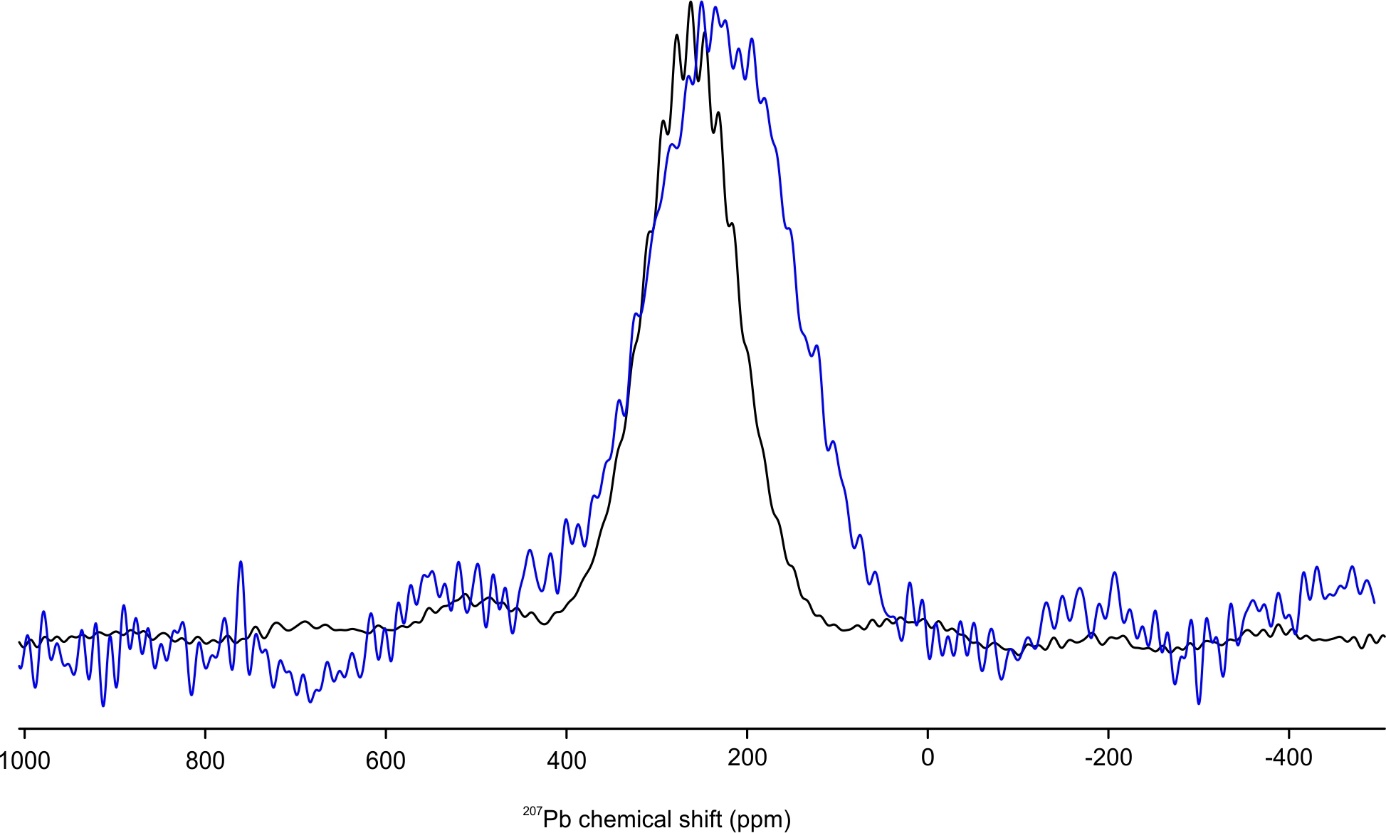


**Figure S3**. ^207^Pb NMR spectra of CsPbBr_3_ acquired on a 16.4 T instrument under static conditions (blue, FWHM = 27 kHz) and 20 kHz MAS (black). The coupling cannot be resolved due to broadening effects under static conditions. The signal shifts slightly due to heating effects under MAS.


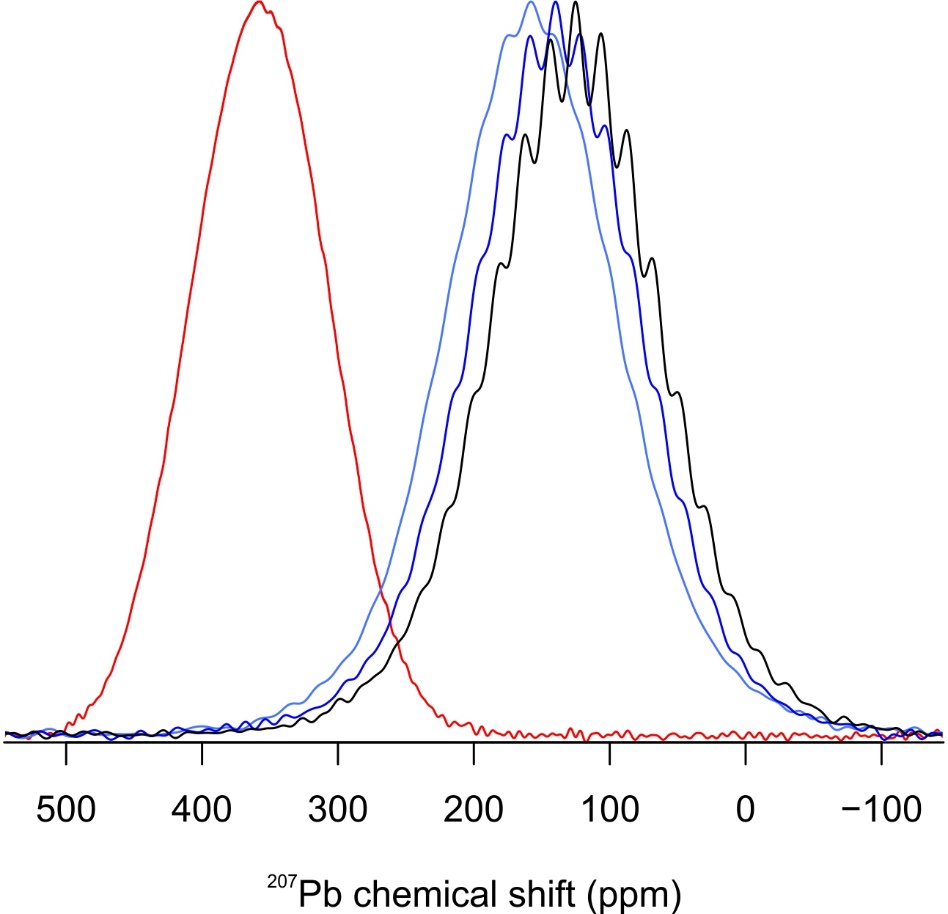


**Figure S4**. ^207^Pb NMR spectra of MAPbBr_3_ acquired on a 14.1 T instrument at different temperatures. RT (red), 141 K (light blue), 120 K (dark blue) and 100 K (black) is shown.


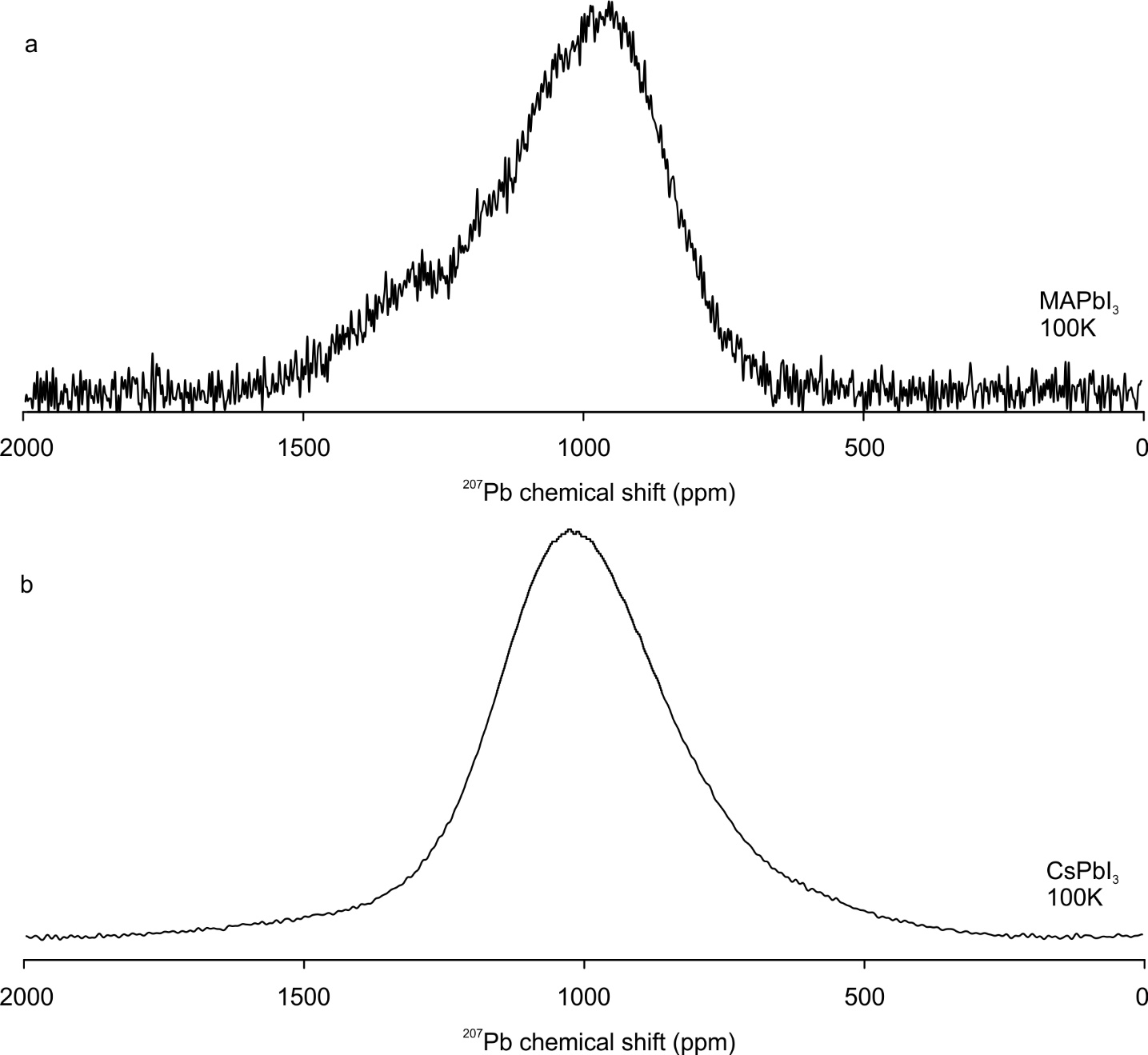


**Figure S5.** 207Pb NMR spectra of (a) MAPbI3 and (b) CsPbI3 at 100 K acquired on a 14.1 T instrument. Both signals show a broad tensor. No coupling can be observed for both compounds. Isotropic chemical shifts are 1030 and 990 ppm, respectively. FWHM were determined to be in the order of 20 kHz for MAPbI3 and 25 kHz for CsPbI3.

**4. XRD patterns**

**Figure S6**. XRD pattern of CsPbCl_3_, CsPbBr_3_, γ-CsPbI_3_, δ-CsPbI_3_ and Cs_4_PbBr_6_ with the calculated patterns shown in negative. The pattern of γ-CsPbI_3_ shows impurities from δ-CsPbI_3_.

**Figure S7**. XRD pattern of MAPbCl_3_, MAPbBr_3_ and MAPbI_3_ with the calculated patterns shown in negative.

**Figure S8**. XRD pattern of FAPbBr_3_ with the calculated pattern shown in negative.

**Table S1.** Structural data of studied APbX_3_ perovskites.

|  | T_c_(K) | Phase | Dimensionality | Methods |
| --- | --- | --- | --- | --- |
| CsPbCl_3_ |  | Triclinic | 3D | EPR^5^ |
|  | 176 | Monoclinic | 3D | EPR^5^ |
|  | 194 | Monoclinic | 3D | EPR^5^, Neutron diffraction^6^ |
|  | 310 | Orthorhombic | 3D | EPR,^5^ ^133^Cs NMR,^7^ ^35^Cl NQR^6, 8, 9^, Sound velocity,^10^ XRD^11^ |
|  | 315 | Tetragonal | 3D | EPR,^5^ ^133^Cs NMR,^7^ ^35^Cl NQR^6, 8, 9^, Sound velocity,^10^ XRD^11^ |
|  | 320 | Cubic | 3D | EPR,^5^ ^35^Cl NQR,^6, 8, 9^ Sound velocity,^10^ XRD^11, 12^ |
|  | | | | |
| CsPbBr_3_ |  | Orthorhombic | 3D | Neutron diffraction,^13^ XRD^12, 14^ |
|  | 360 | Tetragonal | 3D | DSC,^14^ Neutron diffraction,^13^ XRD^12, 14^ |
|  | 406 | Cubic | 3D | DSC,^14^ Neutron diffraction,^13^ ^81^Br NQR,^15, 16^ XRD^12, 14^ |
|  | | | | |
| CsPbI_3_ |  | Orthorhombic | 1D | XRD^12, 17^ |
|  | 580 | Cubic | 3D | XRD^12, 18^ |
|  | | | | |
| MAPbCl_3_ |  | Orthorhombic | 3D | Calorimetry,^19, 20^ IR^19^ |
|  | 172 | Tetragonal | 3D | Calorimetry,^19, 20^ IR^19^ |
|  | 177 | Cubic | 3D | Calorimetry,^19, 20^ IR^19^, ^2^H / ^14^N NMR^21^ |
|  | | | | |
| MAPbBr_3_ |  | Orthorhombic | 3D | Calorimetry,^19, 20^ IR^19^ |
|  | 149 | Tetragonal | 3D | Calorimetry,^19, 20^ IR^19^ |
|  | 154 | Tetragonal | 3D | Calorimetry,^19, 20^ IR,^19^ ^2^H / ^14^N NMR^21^ |
|  | 236 | Cubic | 3D | Calorimetry,^19, 20^ IR,^19^ ^2^H / ^14^N NMR^21^ |
|  | | | | |
| MAPbI_3_ |  | Orthorhombic | 3D | Calorimetry,^19, 20^ IR,^19^ Neutron diffraction^22^ |
|  | 162 | Tetragonal | 3D | Calorimetry,^19, 20^ IR,^19^ Neutron diffraction,^22^ ^2^H / ^14^N NMR^21^ |
|  | 330 | Cubic | 3D | Calorimetry,^19, 20^ IR,^19^ Neutron diffraction,^22^ ^1^H / ^2^H / ^13^C / ^14^N NMR,^21, 23, 24^ ^127^I NQR^24^ |
|  | | | | |
| FAPbBr_3_ |  | Orthorhombic | 3D | XRD^25^ |
|  | 140 | Tetragonal | 3D | XRD^25^ |
|  | 270 | Cubic | 3D | XRD^25, 26^ |
|  | | | | |
| FAPbI_3_ |  | Hexagonal | 1D | XRD^27^ |
|  | 406 | Trigonal | 3D | XRD^27^ |

**5. References**

1. Wells H. L. Über die Cäsium- und Kalium-Bleihalogenide. *Z. anorg. allg. Chem.* **3**, 195-210 (1893).

2. Nazarenko O., Yakunin S., Morad V., Cherniukh I., Kovalenko M. V. Single Crystals of Caesium Formamidinium Lead Halide Perovskites: Solution Growth and Gamma Dosimetry. *NPG Asia Mater.* **9**, e373 (2017).

3. Krieg F.*, et al.* Colloidal CsPbX_3_ (X = Cl, Br, I) Nanocrystals 2.0: Zwitterionic Capping Ligands for Improved Durability and Stability. *ACS Energy Lett.* **3**, 641-646 (2018).

4. Nazarenko O.*, et al.* Guanidinium-Formamidinium Lead Iodide: A Layered Perovskite-Related Compound with Red Luminescence at Room Temperature. *J. Am. Chem. Soc.* **140**, 3850-3853 (2018).

5. Cohen M. I., Young K. F., Chang T. T., Jr. W. S. B. Phase Transitions in CsPbCl_3_. *J. Appl. Phys.* **42**, 5267-5272 (1971).

6. Hidaka M., Okamoto Y., Zikumaru Y. Structural Phase Transition of CsPbCl_3_ below Room Temperature. *Phys. Status Solidi A* **79**, 263-269 (1983).

7. Armstrong R. L., Lourens J. A. J., Stroud J. D. ^133^Cs Spin-Lattice Relaxation Study of Phase Transitions in CsPbCl_3_. *Phys. Rev. B* **13**, 5099-5101 (1976).

8. Armstrong R. L. Pure Nuclear Quadrupole Resonance Studies of Structural Phase Transitions. *J. Magn. Reson. (1969-1992)* **20**, 214-231 (1975).

9. Tovborg‐Jensen N. NQR Investigation of Phase Transitions in Cesium Plumbochloride. *J. Chem. Phys.* **50**, 559-560 (1969).

10. Hirotsu S., Suzuki T. Elastic Constants and Thermal Expansion of CsPbCl_3_. *J. Phys. Soc. Jpn* **44**, 1604-1611 (1978).

11. Ohta H., Harada J., Hirotsu S. Superstructure and Phase Transitions in CsPbCl_3_. *Solid State Commun.* **13**, 1969-1972 (1973).

12. Møller C. K. Crystal Structure and Photoconductivity of Cæsium Plumbohalides. *Nature* **182**, 1436 (1958).

13. Hirotsu S., Harada J., Iizumi M., Gesi K. Structural Phase Transitions in CsPbBr_3_. *J. Phys. Soc. Jpn* **37**, 1393-1398 (1974).

14. Rodová M., Brožek J., Knížek K., Nitsch K. Phase Transitions in Ternary Cesium Lead Bromide. *J. Therm. Anal. Calorim.* **71**, 667-673 (2003).

15. Sharma S., Weiden N., Weiss A. Phase Transitions in CsSnCl_3_ and CsPbBr_3_ An NMR and NQR Study. In: *Z. Naturforsch. A*) (1991).

16. Volkov A. F., Venevtsev Y. N., Semin G. K. Nuclear Quadrupole Resonance (NQR) of ^79^Br and ^81^Br in Perovskite and Orthorhombic Forms of CsPbBr_3_ and CsPbJ_3_. *Phys. Status Solidi B* **35**, K167-K169 (1969).

17. Møller C. K. *The Structure of Cæsium Plumbo Iodide CsPbI_3_*. Munksgaard (1959).

18. Trots D. M., Myagkota S. V. High-Temperature Structural Evolution of Caesium and Rubidium Triiodoplumbates. *J. Phys. Chem. Solids* **69**, 2520-2526 (2008).

19. Onoda-Yamamuro N., Matsuo T., Suga H. Calorimetric and IR Spectroscopic Studies of Phase Transitions in Methylammonium Trihalogenoplumbates(II). *J. Phys. Chem. Solids* **51**, 1383-1395 (1990).

20. Knop O., Wasylishen R. E., White M. A., Cameron T. S., Oort M. J. M. V. Alkylammonium Lead Halides. Part 2. CH_3_NH_3_PbX_3_ (X = Cl, Br, I) Perovskites: Cuboctahedral Halide Cages with Isotropic Cation Reorientation. *Can. J. Chem.* **68**, 412-422 (1990).

21. Wasylishen R. E., Knop O., Macdonald J. B. Cation Rotation in Methylammonium Lead Halides. *Solid State Commun.* **56**, 581-582 (1985).

22. Whitfield P. S.*, et al.* Structures, Phase Transitions and Tricritical Behavior of the Hybrid Perovskite Methyl Ammonium Lead Iodide. *Sci. Rep.* **6**, 35685 (2016).

23. Baikie T.*, et al.* A Combined Single Crystal Neutron/X-ray Diffraction and Solid-State Nuclear Magnetic Resonance Study of the Hybrid Perovskites CH_3_NH_3_PbX_3_ (X = I, Br and Cl). *J. Mater. Chem. A* **3**, 9298-9307 (2015).

24. Senocrate A.*, et al.* The Nature of Ion Conduction in Methylammonium Lead Iodide: A Multimethod Approach. *Angew. Chem., Int. Ed.* **56**, 7755-7759 (2017).

25. Schueller E. C.*, et al.* Crystal Structure Evolution and Notable Thermal Expansion in Hybrid Perovskites Formamidinium Tin Iodide and Formamidinium Lead Bromide. *Inorg. Chem.* **57**, 695-701 (2018).

26. Protesescu L.*, et al.* Monodisperse Formamidinium Lead Bromide Nanocrystals with Bright and Stable Green Photoluminescence. *J. Am. Chem. Soc.* **138**, 14202-14205 (2016).

27. Binek A., Hanusch F. C., Docampo P., Bein T. Stabilization of the Trigonal High-Temperature Phase of Formamidinium Lead Iodide. *J. Phys. Chem. Lett.* **6**, 1249-1253 (2015).
